# Toxicological, Chemical, Social, and Economic Challenges Associated with PFAS and Replacement Aqueous Film-Forming Foams (AFFF)

**DOI:** 10.3390/toxics13090732

**Published:** 2025-08-30

**Authors:** William S. Baldwin, Michael S. Bloom, Katy W. Chung, Subham Dasgupta, Marie E. DeLorenzo, Kelly J. Hunt, Peter B. Key, John L. Pearce, Kylie D. Rock, Philip Tanabe, Morgan A. Jacobellis, Melanie M. Garcia, Lisa J. Bain

**Affiliations:** 1 Department of Biological Sciences, Clemson University, Clemson, SC 29634, USA; subhamd@clemson.edu (S.D.); rock5@clemson.edu (K.D.R.); jacobe2@clemson.edu (M.A.J.); mmgarci@clemson.edu (M.M.G.); lbain@clemson.edu (L.J.B.); 2 Department of Global and Community Health, George Mason University, Fairfax, VA 22030, USA; mbloom22@gmu.edu; 3 National Oceanic and Atmospheric Administration (NOAA), National Ocean Service, Charleston, SC 29412, USA; katy.chung@noaa.gov (K.W.C.); marie.delorenzo@noaa.gov (M.E.D.); pete.key@noaa.gov (P.B.K.); philtanabe@gmail.com (P.T.); 4 Department of Public Health Sciences, Medical University of South Carolina, Charleston, SC 29425, USA; huntke@musc.edu (K.J.H.); pearcejo@musc.edu (J.L.P.)

**Keywords:** PFAS, PFOS, bioaccumulation, biodegradation, half-life, forever chemical, regulation, economic, chronic toxicity

## Abstract

Poly- and perfluorinated alkyl substances (PFAS) are a group of chemicals that are widely used, prevalent in the environment, associated with several toxic effects, and often have long half-lives. Their persistence and relevant toxicity are the primary causes of environmental and human health concerns, and they are referred to as “forever chemicals” because of their persistence. Environmental accumulation caused by slow natural biodegradation and subsequent long environmental half-lives leads to bioaccumulation and makes PFAS more likely to be chronically toxic with potential transgenerational effects. Ultimately, it is this persistence that causes the greatest concern because PFAS-contaminated sites need costly remediation techniques, or else the contaminated areas will not be available for proper economic development because of social and economic suppression. Non-PFAS, alternative Aqueous Film Forming Foams (AFFF) that are considered environmentally friendly, are being heavily considered or currently used for fire suppression instead of PFAS-based products. The bioaccumulation and toxicity of alternative AFFF are just starting to be studied. The purpose of this review is to discuss the basic environmental and human health effects of PFAS and alternative AFFF that propel regulatory changes, increase clean-up costs, reduce economic development, and drive the development of novel alternatives.

## 1. Background

On 29 July 1967, the USS Forrestal caught fire, killing 134 sailors and injuring 161 more [1,2]. Improper safety measures and the lack of high-quality fire suppressants were in part to blame. This fire helped usher in the development and use of new Aqueous Film-Forming Foams (AFFF) that contained poly- and perfluorinated alkyl substances (PFAS) [3]. These PFAS-containing AFFF were highly effective at fighting fires and became widely used in multiple other products, but led to widespread environmental contamination due to their persistence and toxicity.

PFAS are synthetic chemicals that have been used widely as surfactants and polymerization aids, non-stick coatings for cookware, food packaging, finishes for clothing, upholstery, and as firefighting foams for approximately ninety years [4,5,6]. PFAS come in multiple lengths with multiple functional groups. These diverse functional groups may include carboxylic acids, sulfonic acids, sulfonamide, iodides, ethers, and phosphonic or phosphinic acids. The two major forms are sulfonates such as perfluorooctane sulfonic acid (PFOS) and carboxylic acids such as pefluorooctanoic acid (PFOA) [6]. Characterization may also be based on the number of carbons in the chain, with six-carbon chains or less typically considered short-chain PFAS and seven carbons or more considered long-chain PFAS. Chain length is important as it is directly correlated to bioaccumulation [6,7]. With more than 7 million PFAS structures [8], many with long chemical half-lives, these ‘forever chemicals’ have raised several human health and environmental concerns, as exposure is widespread [9,10].

Human PFAS exposure remains prevalent for common PFAS such as PFOS, PFOA, and perfluorohexane sulfonic acid (PFHxS) despite U.S. and European manufacturing phase-outs in the early 2000s and listing in the Stockholm Convention [6,11]. Contaminated food and water, household dust, and indoor air continue to be important sources. Furthermore, these chemicals and other PFAS are produced internationally by multiple countries and imported in consumer goods such as firefighting foams, non-stick cookware, stain-resistant products, clothing, mattress pads, table cloths, personal care products, and cleaning products [12], which means the exposure, bioaccumulation, and toxicity risks of PFAS will continue.

**Figure 1:**
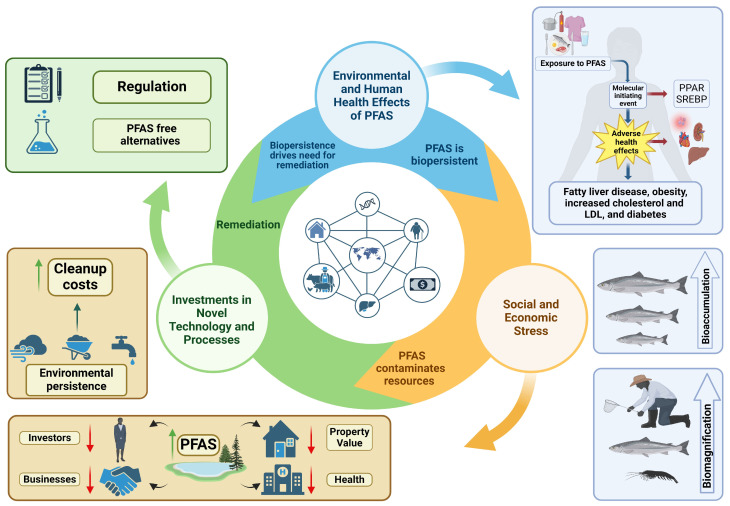
PFAS exposure has negative consequences on the environment and human health, and in turn negative social and economic impacts related to it’s toxicity and persistence. As toxicologists, we often consider only the bioaccumulation or toxic responses; however, the novel properties of PFAS have led to reduced economic investment, repressed property values, expensive remediation and clean-up costs, and ultimately a search for alternatives. Adverse environmental and human health effects caused by PFAS are often the result of biomagnification and bioaccumulation (blue). PFAS’s persistence and toxic effects leads to the need for regulations and novel remediation strategies (green). In addition, there are social and economic stresses caused by PFAS bioaccumulation and toxicity such as increased clean up costs, reduced property value, reduced investment, and increased healthcare costs. This figure was created using Biorender.

The manufacturing and subsequent persistence of PFAS led to significant contamination in several areas or hotspots [13,14]. These hotspots affect human and environmental health, leading to increased healthcare and compliance costs, and adversely impacting property values and economic development due to potential clean-up costs. Therefore, PFAS significantly reduce investment in these regions [15,16,17]. It is the widespread use and persistence of PFAS that lead to downstream adverse effects on human health, the environment, and the economy (Figure 1).

Multiple PFAS, including PFOA, PFOS, and long-chain fluorotelomers, have been used in AFFF as firefighting foams since the 1970s. AFFF are typically used as class B firefighting foams to extinguish gas, oil, and jet fuel fires (flammable liquids, gases, or greases), and therefore are commonly available at airports and military bases [18,19]. The ensuing contamination threatens the health and well-being of the environment, firefighters, and local communities [19]. Therefore, synthetic fluorine-free foams, also known as “F3” foams, that are effective at putting out fires are being developed.

In February 2024, the U.S. FDA announced that PFAS-containing grease-proofing agents that come into contact with food, such as in cookware and food packaging products, are no longer being sold [20]. In April 2024, the United States Environmental Protection Agency (USEPA) established enforceable drinking water limits for PFOS, PFOA, hexafluoropropylene oxide dimer acid [GenX (HFPO-DA)], perfluorononanoic acid (PFNA), and PFHxS, with limits ranging from 4 to 10 parts per trillion (pptr). Specifically, PFOA and PFOS were capped at 4 pptr; GenX, PFNA, and PFHxS were capped at 10 pptr as a Maximum Contaminant Level (MCL). This regulation also includes mixtures of two or more of PFHxS, PFNA, perfluorobutane sulfonic acid (PFBS), and HFPO-DA. Public water systems have until 2027 to complete initial monitoring and until 2029 to implement solutions to reduce these PFAS in accordance with the MCLs [21]. However, in May 2025, the USEPA announced its intent to rescind its drinking water standards for GenX, PFHxS, PFNA, and PFBS. The proposed rule redefining PFAS standards should come into effect in the spring of 2026 [22].

The following review examines PFAS persistence, toxicity, costs, and potential alternatives.

## 2. PFAS Exposure and Bioaccumulation

Decades of widespread use, along with mobility and persistence, have resulted in PFAS contamination worldwide. Recent studies revealed widespread contamination of U.S. municipal drinking water supplies with detectable levels of PFAS, with PFOA and PFOS being most consistently reported [23,24,25]. Importantly, these and other PFAS have been measured in the vast majority of biological samples, such as serum and urine collected from nationally representative studies of U.S. children and adults [26,27]. For example, 97% of human samples contained measurable PFAS in the NHANES study [27,28]; other studies found as many as 100% of samples contained PFAS [28]. Such findings present important public health concerns given bioaccumulation within humans, persistence in the environment, and their continued production and use in many areas [29,30,31]. To further complicate matters, recent studies reveal that exposures do not occur in isolation, but rather as combinations of substances, indicating that real-world exposures to PFAS occur as complex mixtures with unknown biological implications [28,32]. For example, some studies have found that mixtures of PFAS work in a predictable manner that leads to neurotoxicity and cytotoxicity and follow typical concentration-addition mixture type dose–response curves [32]. However, other studies found that specific combinations of chemicals (with both PFAS and other persistent organic pollutants or metals) were more likely to cause adverse outcomes in children [28]. In addition, other studies found that PFOA antagonized PFOS’s ability to form reactive oxygen species and cause acute toxicity [33].

In addition to human exposures, the presence of PFASs in tissues of aquatic and terrestrial species has been documented in studies conducted across every continent, including remote regions far from direct sources of emission, such as the high Arctic, Antarctica, and oceanic islands [34,35]. Further, some PFAS compounds (e.g., PFOS) have the potential to biomagnify with increasing trophic levels in a variety of both freshwater and marine ecosystems [6,35,36,37,38,39,40,41,42,43]. The bioaccumulation of PFAS has been noted to be greater in marine environments compared to terrestrial systems [44]. Some of the greatest accumulations of PFAS have been shown in marine mammals, compared to aquatic animals with gills [45,46]. PFAS accumulation within an organism’s tissues may cause adverse health effects directly, through maternal transfer, and also cause indirect effects to higher-trophic-level predators, including human consumers of fish and shellfish.

Bioaccumulation potential generally increases with PFAS carbon chain length, and sulfonic acids are more bioaccumulative than carboxylic acids of the same carbon chain length [6]. Short-chain carboxylic acids (C < 7) were determined to have log bioconcentration (BCF) values < 1 in fish and thus would not be considered bioaccumulative [44]. Bioaccumulation of both linear and branched isomers of PFOS has been shown in Eastern oysters, but the branched isomers were more readily eliminated than the linear isomers [47]. Our understanding of bioconcentration and bioaccumulation is crucial as biological persistence is still considered the most prominent toxicology issue in marine and aquatic toxicology today, with PFOS and other, mostly legacy, PFAS of highest concern [48].

Typical bioaccumulation models based on chemical partitioning into lipids are not applicable to most PFAS, in part due to their lipophobic properties and in part due to their affinity for proteins that bind to lipids, making lipid partitioning models inaccurate [49,50]. Fatty acid-binding proteins (FABPs), serum albumin, peroxisome proliferator-activated receptors (PPARs), organic anion transporters (OATs), and organic anion transport proteins (OATPs) are some of the proteins that bind PFAS. Of these, FABPs, serum albumin, and OATs are involved in PFAS’s bioaccumulation [51]. FABPs bind PFAS with high affinity, and the greater the induction and binding to FABP and serum albumin, the greater the half-life [52,53,54,55,56,57]. PPARs are nuclear receptors that respond to fatty acids and modify lipid uptake, metabolism, and storage, and therefore regulate liver lipid accumulation [58,59,60,61,62].

Both albumin and FABPs bind numerous fatty acids in addition to PFASs. FABPs even change conformation upon binding [51]. The Posterior Inclusion Probabilities (PIPs) obtained from molecular descriptor analysis revealed that molecular flexibility, atomic polarizabilities, and the number of carbon atoms were the primary molecular characteristics that led to binding of PFASs to (liver) L-FABP [56]. Species differences in L-FABP binding were also noted, with human, rat, and rainbow trout having stronger binding affinities than Japanese medaka and fathead minnow [63]. The number of carbons may also have an effect, as eight-carbon compounds increased lipid synthesis more, while ten-carbon PFAS decreased lipid excretion [64]. Albumin binds PFAS at multiple positions, but does not change conformation as FABP does [51]. Serum albumin binds PFAS in the blood, inhibiting filtration and excretion by the kidneys [51]. In summary, L-FABP helps transport and keep PFASs in the liver where they preferentially bioaccumulate. The liver and serum are typically the tissues with the greatest bioaccumulation because of PFAS binding to FABP and albumin [6,51,56].

In addition to binding proteins, OATs and OATPs also enhance bioaccumulation. The four proteins most associated with bioaccumulation in the liver due to enterohepatic circulation are L-FABP, serum albumin, apical sodium-dependent bile acid transporter (ASBT), and sodium taurocholate co-transporting polypeptide (NTCP) [65,66] as NTCP transports PFAS to the bile but ASBT reabsorbs them from the intestines. Once PFAS are absorbed, most are excreted through the kidney. However, reabsorption is common through OATP1A1 as well as OATP1B3 and OATP2B1 [67]. Some species and sex differences [45,68] in bioaccumulation are likely caused by transporters. The half-life of PFOA is only hours in female rats, but several days in male rats [69], probably because of reduced expression of OATP1A1 in female rats [70]. The role of transporters in bioaccumulation is not fully understood. For example, L-FABP protein and *fatp1* gene expression were associated with differences in liver PFOS retention between Cyp2b-null and hCYP2B6-Tg mice [53]; however, the role of *fatp1* in PFOS liver absorption has not been studied to our knowledge.

Exposure to mixtures of PFAS will also influence uptake, binding, transformation, and toxicity. In fish, differential protein-binding interactions between PFOS and PFOA were observed, where PFOS did not upregulate peroxisome proliferator-activated receptor alpha (*pparα*) but did significantly upregulate a downstream product, apolipoprotein A4 (*apoa4*) [33]. PFOA had the opposite binding pattern and resulted in decreased toxicity when mixed with PFOS [33].

Environmental exposure conditions such as salinity and temperature may also influence bioavailability and resulting toxicity of PFAS compounds. Chung et al. [71] reported that PFOS toxicity was greater at higher temperatures for *C. variegatus*, and greater at lower salinities for grass shrimp (*Palaemon pugio*) and eastern mud snail (*T. obsoleta)*.

## 3. Bioanalytical Challenges for Identification and Quantitation of PFAS

Exposure studies, both in vivo and in vitro, require quantitation of dosed PFASs. Biomonitoring studies aim to confidently identify and quantify PFASs that are present in a biological matrix. However, PFAS identification and quantitation are not straightforward. Not only are PFAS ubiquitous, but the number of chemicals classified as PFAS is as high as 7 million [8]. Other factors such as the physiochemical properties of PFAS, the lack of standard methods, the need for low detection limits, the presence of analytical interferences, and the ability to biotransform can lower confidence in correct identification and accurate quantitation. Few standard methods are available for the detection and quantitation of PFAS, and several that are available are intended for aqueous matrices [72]. Quantitation can be difficult due to the lack of appropriate, PFAS-free control matrices, limited commercial availability of analytical standards and reference materials, and paucity of commercial isotope-labeled internal standards.

Quantities of biological samples are often small and prevent accurate quantitation of PFAS that are present at low concentrations. This is especially true for human biomonitoring, for which both sample volumes are minimal and PFAS concentrations are typically close to detection limits. PFAS exposure can occur through multiple pathways [73], which makes the selection of appropriate models for understanding potential health effects challenging, as does the variety of biological systems impacted by PFAS. Due to the ubiquitous nature of PFAS, care must be taken to minimize and avoid contaminated laboratory animal feed that could influence a toxic response as well as contribute to internal dose measurements for exposure studies [74,75].

Further complicating the accurate quantitation of PFAS in biological matrices is the ubiquitous nature of PFAS in common laboratory materials. Care must be taken to minimize and avoid contamination of samples from typical analytical chemistry laboratory sources such as solvents, water, plasticware including pipette tips and 96-well plates, glassware, filters, foil, vials, septa, manifold lids, nitrogen evaporation needles, and analytical instrumentation. PFAS adhere to both glass and plastics [76], which leads to often conflicting advice on the use of labware such as recommendations to avoid using glass [77,78], only use polypropylene (PP) [79], use polystyrene (PS) but not PP [80], use glass instead of PP, PS, and polycarbonate [76], and avoid use of polytetrafluoroethylene (PTFE) [5]. Filters used during sample collection and preparation have been shown to contain PFAS that can contaminate samples or bind and remove PFAS from the sample [81,82]. Instrument manufacturers offer specialized kits and parts to reduce PFAS contamination that occurs during separation and/or mass spectrometry analysis due to PFAS-containing instrument components. Best practices for laboratories determining PFAS include testing all solvents and supplies for PFASs prior to use on samples, replacing solvent lines and seals on liquid chromatographs with PEEK tubing and other PFAS-free substitutes, use of an in-line delay column, use of blanks to monitor contaminants, and frequently cleaning mass spectrometer sources.

Some PFAS have properties including varied solubility and air sensitivity that may make them difficult to handle in the laboratory [83]. PFAS may sublime [8], leach into extracts during sample preparation [84], or decompose during storage or dosing [85]. Control matrices used for the preparation of calibration curves and quality control samples may also be a source of potential contamination. Fetal bovine calf serum is used by some researchers as a control matrix due to the assumption that it is PFAS-free [24,86]. In other instances, solvent-based standards may be used due to the lack of availability of an appropriate PFAS-free control matrix [87,88].

Detection of PFAS in biological matrices is hampered by the lack of published methods. Few standard methods are available for the detection of PFAS, and they are only applicable to a small number of semi-volatile compounds [72,75,89,90]. For example, most standard USEPA analytical methods for the detection of PFAS are validated for aqueous samples [72], target fewer than 25 common PFAS, and employ liquid chromatography–tandem mass spectrometry (LC-MS/MS) performed on triple-quadrupole mass spectrometry systems. A few standard methods are available for use with biological matrices such as cells, cell media, biofluids, or tissues that are relevant to toxicity studies. The USEPA Method 1633 expands detection to 40 PFAS in fish tissue, along with environmental matrices [91]. A worldwide inter-laboratory assessment of targeted analyses of 22 PFAS in matrices including human milk and plasma demonstrated that PFAS measurements between laboratories are comparable [90]. The U.S. Center for Disease Control and Prevention (CDC) uses an on-line solid-phase extraction (SPE) method for the analysis of 17 PFAS in human serum [92]. Researchers may be able to modify standard methods to fit with the matrices they are interested in, such as human plasma, rodent liver and other organs, milk, and zebrafish embryos. But in many instances, new analytical methods must be developed and validated.

A literature search using Abstract Sifter with terms including PFAS, rat liver, plasma, and mass spectrometry yields hundreds of articles, most related to the detection of PFAS in human matrices for biomonitoring [93]. An LC-MS/MS method with matrix-specific sample preparation procedures was used for the detection and quantitation of PFAS in milk from healthy human mothers, as well as liver samples collected postmortem from human donors [87]. A review of analytical methods for the detection and quantitation of PFAS in human matrices provides additional examples [94]. Other recent publications describe LC-MS/MS methods for the determination of PFAS in rat livers and plasma [95,96,97]. Huang et al. [98] included methods for the detection of PFAS in rat plasma, liver, kidney, and brain for a toxicokinetics study. The detection of PFAS and glucuronide metabolites was documented in a recent study [99]. Toxicokinetic studies have made use of both LC-MS/MS and gas chromatography–tandem mass spectrometry for detection and quantitation of PFASs in plasma, serum albumin, and hepatocyte media [55,99,100].

Analytical interferences from endogenous chemicals in biomatrices can lead to false identifications or miscalculation of PFAS concentrations [101]. Steroid sulfates have common tandem mass spectrometry transitions with perfluorohexane sulfonic acid (PFHxS) that can lead to false identifications or overestimation of concentrations in human serum unless alternate transitions were selected, high-resolution mass spectrometry was employed, or the compounds were chromatographically separated [102]. Similarly, taurodeoxycholic acid and perfluorooctanesulfonic acid (PFOS) share a common transition that could confuse identification or quantitation in biomatrices [103]. An interference in a chemical standard of perfluoro-3,5,7,9,11-pentaoxadodecanoic acid (PFO5DoA), a novel PFAS observed in human blood, was identified by Kotlarz after it had contributed to an overestimation of concentrations in human serum [24].

PFAS are commonly known as “forever chemicals”, but some including perfluorosulfonamides and fluorotelomers are able to biotransform to generate metabolites [104]. For example, PFAS-based fluorotelomer alcohols (FTOH) biotransform to perfluorocarboxylic acids such as PFOA and perfluorohexanoic acid (PFHxA). The Total Oxidizable Precursors (TOP) assay can be used to detect FTOH as it oxidizes the unknown precursors and intermediates into stable PFAS [105]. Because of concerns surrounding precursor chemicals, Europe is considering adding FTOH 6:2 and FTOH 8:2 to the regulatory sum of PFAS of primary concern in drinking water [106].

Other examples of dosing studies with precursor compounds that yield metabolites that may be toxic include perfluorohexanesulfonamide (PFHxSA), which is known to metabolize to PFHxS [104]. PFHxS is highly toxic and included in a USEPA national drinking water standard [107]. Exposure studies evaluating the toxicity of PFHxSA may assume toxic effects are related to PFHxSA, while being unaware of the presence of PFHxS. Other recent studies have observed biotransformation of dosed PFAS compounds [99,100,108]. Biomonitoring studies may detect metabolites at higher concentrations than chemical precursors [109] or may use targeted methods that do not include precursors. Incorporation of nontarget analysis (NTA) using high-resolution/accurate mass LC-MS/MS into study designs may provide insights into the full distribution of PFAS present in biomatrices [110,111]. Another NTA method that may prove useful is Combustion Ion Chromatography (CIC), which quantifies total organofluorine content after high-temperature combustion [112]. It is complementary to LC-MS/MS; however, it does not identify individual PFAS.

## 4. PFAS and the One Health Concept

The One Health concept aims to balance and optimize the health of humans, animals, and ecosystems [113]. In the field of toxicology, One Health recognizes that shared environments allow potential exposure to the same toxic agents and chemical-associated health risks. This approach is particularly relevant for studies of PFAS exposure and toxicity [48,114,115,116,117,118,119]. In the context of One Health, biomonitoring studies have demonstrated that shared environments, food, and water sources can contribute to similarities in human and animal PFAS exposure profiles. Recent results demonstrate similar adverse system toxicology effects across taxa and even common toxicity themes across PFAS classes [120,121]. Key findings from these studies include differences in spatial and temporal trends in PFAS contamination and exposure, the prevalence of PFAS within food webs, including livestock and crops for human consumption, and overlap in adverse health effects between humans and animals, including three primary targets of PFAS toxicity: the immune system, kidneys, and liver [73,119,122,123,124,125,126,127,128,129].

## 5. Human Health Effects and Systemic Toxicity of PFASs

Improved understanding of the health effects of PFAS is a current research priority for epidemiologists in the environmental health research community. Epidemiological studies have shown that exposures to some PFAS have been associated with a wide variety of health effects, including immune suppression, high cholesterol, liver toxicity, fetal growth restriction, and certain types of cancer [6,73,130,131]. Results from observational/epidemiological studies in human populations are mixed; however, continued research is yielding greater health concerns [132,133]. PFAS cross the placental barrier, resulting in fetal exposure [134], with potential developmental sequelae. Recent meta-analyses of studies in human populations have reported lower birth weight and birth length associated with greater PFAS exposure, as well as greater risks of preterm birth and small-for-gestational-age infants [135,136]. A greater risk of thyroid disease and altered thyroid hormone levels has also been reported in human studies of PFAS exposure in adults and children [137,138], which could have a myriad of downstream adverse health impacts. However, contradictory study results and some methodological concerns also preclude a causal connection at present [139,140]. Other evidence from human studies suggests adverse effects on metabolism, including diabetes, lipid homeostasis, body composition/obesity, and vascular health, in association with greater gestational and postnatal PFAS exposure, but again with mixed results [141,142,143]. Less common PFAS [144], replacement PFAS [145] and PFAS mixtures [146] are also areas of concern, but less research has been performed compared to legacy PFAS.

Toxicological evidence is growing as results from a number of experimental studies, both in vitro and in vivo, indicate adverse human health effects from PFASs [147,148]. Several PFASs and their replacements have been identified as endocrine-disrupting chemicals (EDCs) in experimental studies, altering the function of sex steroid and thyroid hormones [97,137,149,150]. Other studies show binding to PPARs, with potential developmental and metabolic effects [151,152,153]. Furthermore, a U.S. National Toxicology Program literature review identified PFOA and PFOS as human immune hazards [154], and a collaboration of industry and academic experts called for further investigation [155].

PFAS cause a large number of negative effects on multiple organ systems. Toxicology studies in animal models, cell models, and key molecular initiating events such as PPARα and sterol regulatory element-binding protein (SREBP/SREBF) activation provide mechanisms for PFAS-mediated metabolic diseases such as fatty liver disease, obesity, increased cholesterol and LDL, and diabetes [6,52,60,68,145,156,157].

**Figure 2:**
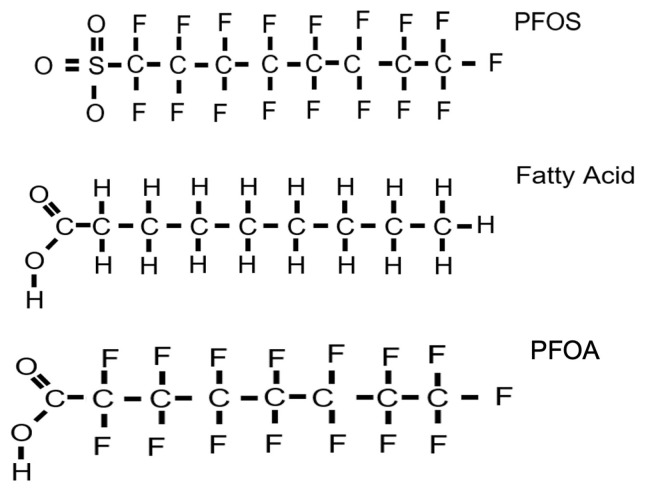
The structure of many PFAS shows high similarity to fatty acids, with the replacement of hydrogens with fluorines. An eight-carbon mid-chain fatty acid is compared to PFOS and PFOA.

Both legacy and alternative PFAS appear to activate similar pathways [60,145]. For example, a recent toxicogenomics study indicates that most legacy and alternative PFAS activate PPARα; many also activate SREBP, constitutive androstane receptor (CAR), and nuclear factor erythroid 2-related factor 2 (NRF2), and some inhibit signal transducer and activator of transcription 5b (STAT5b) [60]. These key molecular initiating events with PPARα activation are central to the disruption of most pathways associated with obesity, diabetes, high cholesterol, and other metabolic-dysfunction-associated fatty liver diseases (MAFLDs). The large overlap between legacy and alternative PFAS is interesting. Evidence from epidemiology studies confirms most if not all of the disease results (see below), including studies that demonstrate an association between concentrations of PFAS or PFAS-containing mixtures in mothers and childhood obesity [28,142,158,159]. Overall, it is not surprising that PFAS bind albumin, fatty acid-binding proteins (FABPs), and PPARs, and disrupt energy signaling given their similar structure to short- and mid-chain fatty acids (Figure 2) depending on the length of the PFAS [56,160].

New shorter-living, typically short-chain PFAS and PFAS-forming formulations have been tested recently. There is still much to be learned about the bioaccumulation profiles of these PFAS-containing compounds, including PFAS-containing AFFF. In addition, they are not as strictly regulated as legacy PFAS, as total PFAS in drinking water can legally reach 0.5 μg/L [161,162]. A recent study comparing legacy, short-chain, and precursor PFAS [i.e., substances that can transform into perfluoroalkyl acids (PFAAs)] that form PFOS in primary human hepatocytes found that the replacement short-chain PFAS and precursor PFOS were more potent at inducing steatosis and lipogenic gene expression than legacy PFAS [145]. PFAS precursors are also likely to add to the total PFAS accumulation in human tissues; however, their role in bioaccumulation is not known.

Other adverse health effects include endocrine disruption, some of it mediated through nuclear receptors, increased oxidative stress, neurotoxicity, reproductive and developmental toxicity, pulmonary toxicity, and cancer, of which some of these effects may be elicited through reduced immune surveillance [6]. For example, PFAS levels have been associated with cancer in humans, and PFAS have been shown to increase cancer incidence in laboratory animals [131,163,164,165]. In the Danish population, the greater the level of PFAS, especially PFOA and PFOS, the greater the risk of cancer [166]. Concentrations of PFOS, PFOA, PFNA, and PFHxS are inversely associated with income [167]. In addition, non-Hispanic Black populations may be exposed to more PFAS and have higher serum levels of some PFAS than other racial and ethnic groups, and this may manifest itself in increased cancer risk and other diseases such as hypertension [164,168,169,170,171].

The long half-lives of many PFAS allow for bioaccumulation and the time necessary for transgenerational effects to occur. PFAS have been associated with reproductive problems and developmental delays [96,134,172] that in part may be due to the disruption of PPARγ signaling [173]. Other toxicity and health issues include cardiovascular disease, hypothyroidism, neurotoxicity, and many other adverse effects associated with human PFAS exposure in agreement with PFAS toxicity assessments performed in laboratories [154,174,175,176,177,178,179]. Immunotoxicity [154,180] and reduced responses to vaccines have also been measured [181]. In summary, there are a multitude of potential adverse health effects that might be caused by PFAS exposure based on laboratory evidence and epidemiological studies.

**Figure 3:**
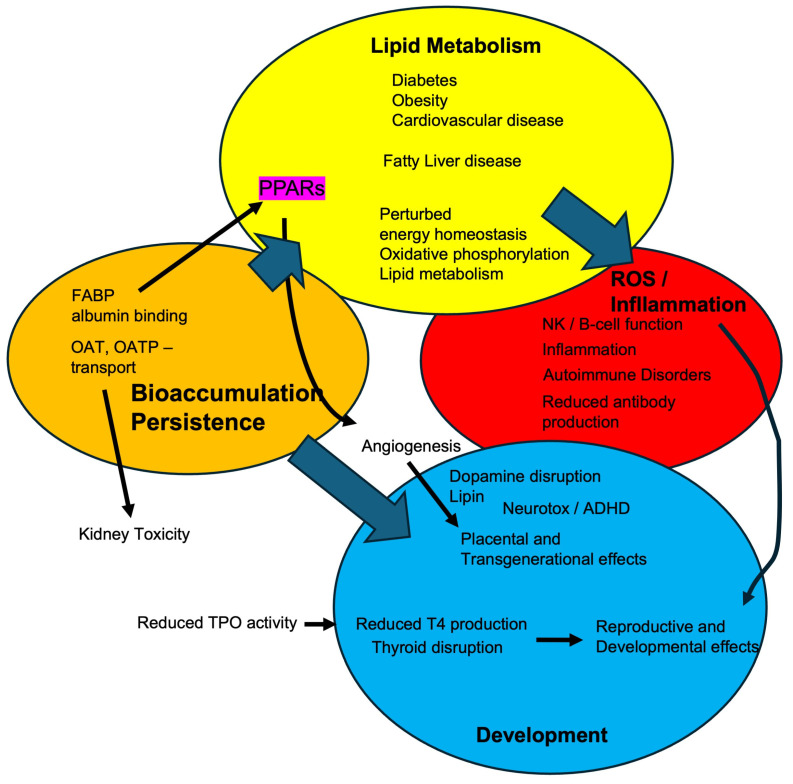
PFOS, PFOA, and other PFAS have multiple effects on several different organ systems. Most of these adverse effects are complicated or worsened by the bioaccumulation of some PFAS. This allows for disruption of lipid metabolism and other metabolic pathways, subsequent perturbations in reactive oxygen species and inflammation, and development, including neurodevelopment, and immune surveillance.

Figure 3 is a summary figure presenting some possible molecular initiating mechanisms of PFASs and their ultimate systemic effects. Many of PFASs’ negative effects are linked to their environmental persistence and bioaccumulation, which is also stressed in Figure 3. OATs and OATPs increase the retention of PFAS in the kidney due to tubular reabsorption [53,67,69,182]. This can also lead to kidney toxicity and cancer [54,70,183,184,185,186]. Proteins such as serum albumin and FABP bind PFOA, PFOS, and other PFASs and increase their half-lives [56,160,187]. L-FABP also helps transport PFASs to the PPARs, where they may activate PPARs such as PPARα or potentially inhibit PPARγ [56,188]. Disruption of PPAR activity is associated with metabolic disease following PFAS exposure, including altered mitochondrial function, changes in lipid metabolism, and fatty liver disease. These effects may lead to obesity, diabetes, and cardiovascular disease [6,52,60,159,189,190,191,192,193].

In addition to metabolic changes, PPARγ disruption by PFASs alters angiogenesis and placental function [173,193,194]. In turn, PFAS exposure can lead to developmental effects in children from exposed mothers, including neurotoxicity, immune surveillance issues, and childhood obesity [96,172,195]. Immune suppression may occur through multiple pathways. PFAS can disrupt PPARs and NF-kB, perturb fatty acid metabolism, or induce oxidative stress (reactive oxygen species/ROS) [53,196,197,198]. These molecular events can cause changes in cytokine release, B-cell, T-cell, and natural killer cell functions [196,199,200] that lead to repression of antibody production, increased asthma, autoimmune disease, and a reduced response to vaccines [196,199,201,202,203]. The nervous system and thyroid are also targets. Enzymes such as thyroid peroxidase (TPO) or tyrosine hydroxylase are inhibited or down-regulated, leading to reduced thyroid hormone or dopamine release and signaling [137,204], which can lead to alterations in neurodevelopment, neurotoxicity [205], and recently reported increases in Attention Deficit/Hyperactivity Disorder (ADHD) and potentially autism [206,207].

## 6. Regulation of PFAS

In the United States, the Environmental Protection Agency (EPA) has the authority to regulate PFAS at the national level through the Toxic Substances Control Act (TSCA), Comprehensive Environmental Response, Compensation, and Liability Act (CERCLA), and the Safe Drinking Water Act (SDWA) [208]. Owing to concerns regarding the persistence and toxicity of long-chain PFASs, the EPA began regulating these chemicals in 2002 with Significant New Use Rules (SNURs) requiring manufacturers to provide advanced notification about the production or import of perfluorooctane sulfonyl fluoride (POSF) and some of its salts and homologs [208,209,210,211]. Shortly thereafter, in January 2006, the EPA implemented the 2010/2015 PFOA Stewardship Program [212]. This program invited eight major companies in the PFAS industry to commit to reducing PFOA in factory emissions and products by 95% by 2010 and to work toward eliminating PFOA from emissions and products by 2015. All eight companies, which operate globally, committed to this stewardship program and to working towards a global phase-out of PFOA and related chemicals. In conjunction with these phase-outs, the fluorochemical industry has introduced replacement chemistries, prioritizing shorter-chain PFAS and perfluoroalkyl ether moieties, claiming they are less bioaccumulative and toxic [209,213]. Through the SDWA, the EPA continues to monitor concentrations of PFAS in drinking water supplies and has made modifications to lifetime drinking water health advisories in response to accumulating evidence of toxicity [213]. These modifications include reducing the health advisory levels for PFOS and PFOA, from 70 parts per trillion (pptr) established in 2016 to 0.02 pptr PFOS and 0.004 pptr PFOA in 2022, and establishing health advisory levels for some of the replacement chemistries, including HFPO-DA (also known as GenX) and PFBS. Health advisory levels are not enforceable standards; however, the EPA finalized a National Primary Drinking Water Regulation (NPDWR) to establish legally enforceable concentrations, known as MCLs, for six PFAS in drinking water in 2024 [162]. Note however that in 2025, the EPA proposed that it will keep the MCLs only for PFOA and PFOS and rescind or reconsider the limits for PFHxS, HFPO-DA, PFNA, and mixtures of these with PFBS (https://www.epa.gov/newsreleases/epa-announces-it-will-keep-maximum-contaminant-levels-pfoa-pfos, accessed on 18 June, 2025) [214]. With the vast structural diversity of PFAS, consisting of many thousands to millions of different compounds, regulations could benefit from a change in strategy from the current approach of regulating a single compound and related substances to regulating large groups of PFAS based on chemical class (i.e., physiochemical, environmental, and toxicological properties) [209,215]. This is especially true of PFAS because while six PFAS are tightly regulated in drinking water, total PFAS concentrations of non-legacy compounds can reach up to 0.5 μg/L in drinking water, probably due to uncertainty in their bioaccumulation and human toxicity.

## 7. Impacts of PFAS on Economic Development, Business, and Property Value

PFAS are also of concern due to their potential economic impacts. Companies involved in the manufacturing or use of PFAS-containing products may encounter legal responsibilities and lawsuits initiated by affected individuals, communities, and government entities seeking compensation for health issues and property harm resulting from PFAS contamination. Notably, compensation in some states in the U.S., such as Minnesota (USD 850 million), Alabama (USD 39 million), and Michigan (USD 168 million), highlights the substantial legal costs associated with defending against or resolving such litigations, presenting a considerable financial burden to state or federal agencies in addition to the companies involved [15]. Conversely, state and local governments may also face lawsuits from manufacturers due to PFAS reduction policies and health regulations related to drinking water. In addition to direct liability and legal costs, companies associated with PFAS contamination may face reputational damage, leading to reduced sales and market share.

Growing concerns about PFAS may result in restrictions or bans on international trade in products containing PFAS. For instance, a report released by the US Chamber of Commerce in September 2023 revealed that the economic and fiscal impacts of PFAS-containing goods exported from the United States to the European Union amounted to USD 314 billion in value and over 500,000 jobs [216]. These could be jeopardized if the European Union enforces its proposed PFAS ban from February 2023. Consequently, these regulations can impact industries reliant on the global supply chain for PFAS-containing materials and products.

**Figure 4:**
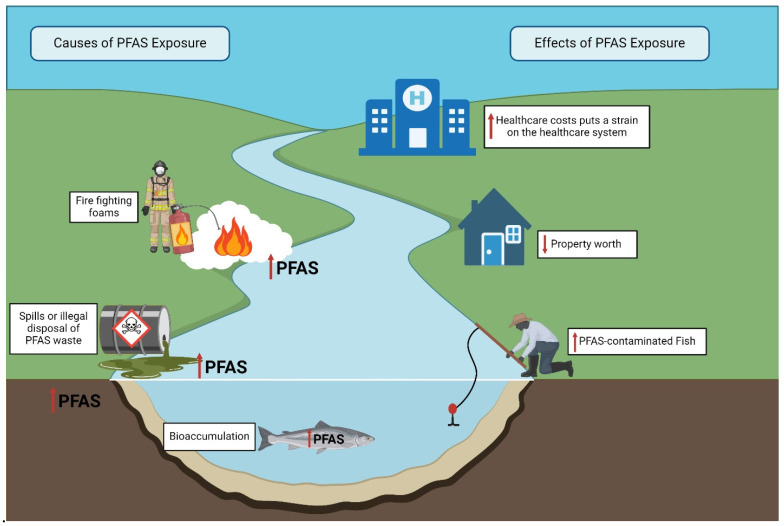
PFAS contamination has negative consequences on property values and healthcare costs. These problems are especially acute in poor or minoritized areas where factories, landfills, testing grounds, and waste facilities are more likely to be found. This figure was created with Biorender.

Moreover, PFAS contamination can dissuade potential investors, businesses, and residents from considering locations affected by contamination, potentially negatively affecting the economic growth and overall economic well-being of a region [16]. Properties located in proximity to PFAS-contaminated sites may witness a decline in their worth (Figure 4). Research from Australia demonstrated that property values in Williamtown, New South Wales, an area contaminated with PFAS, fell by 15% compared to the current market value had contamination not existed [17,217]. This can result in financial losses for property owners and local governments, along with potential reductions in property tax revenues. This may be even more so in some minority communities where PFAS contamination is greater due to local factories and municipal pollution sources [12,218,219].

## 8. PFAS and Healthcare Costs

The health repercussions of PFAS exposure can lead to escalated healthcare costs, as individuals affected may require medical treatment and monitoring. As an illustration, a study assessed healthcare expenses in Nordic countries for populations impacted by PFAS exposure through occupation, drinking water, or background exposure. According to this study, communities exposed to PFAS in their drinking water due to proximity to chemical plants or other industries incurred estimated annual healthcare costs ranging from EUR 2.1 to 2.4 million [16]. These expenses can place a strain on healthcare systems and insurance providers.

Finally, the insurance sector may face increased costs and losses due to PFAS-related claims, as indicated in a report from the Hinshaw Law Firm [220]. These can subsequently translate into higher insurance premiums for businesses and individuals.

To tackle these economic challenges, governmental bodies, corporations, and local communities are collaborating to institute regulations, execute clean-up initiatives, and innovate new technologies to reduce the adverse effects of PFAS contamination and the costs linked to it. Nonetheless, the economic consequences of PFAS contamination are intricate and enduring, demanding continued dedication and resources to effectively address the issue.

## 9. Clean-Up and Compliance Costs Due to PFAS

Of course, clean-up costs are also expensive. One of the major economic challenges associated with PFAS concerns the costs incurred in the remediation of contaminated sites, encompassing both surface and groundwater. PFAS have prolonged environmental persistence, resulting in costly and time-consuming efforts to cleanse polluted soil, water, and air. For instance, the endeavor to bring drinking water in Orange County, California, to the state’s recommended PFAS levels is projected to amount to at least USD 1 billion [15]. In March 2023, the USEPA introduced new regulations under the SDWA for PFAS, entailing total annualized expenses ranging from USD 772 million to USD 1.2 billion [221]. These fiscal responsibilities can be borne by state and federal agencies, as well as the public through utility costs. Additionally, these requirements can impose compliance costs on local and smaller businesses, resulting in investments in novel technologies and processes to minimize PFAS emissions and contamination. This has been an impetus for the development of alternatives to PFAS.

## 10. Economic Impacts Lead to PFAS-Free Alternatives—AFFF as an Example

As mentioned previously, the untimely death of 134 sailors and more injured in July 1967 on the USS Forrestal led to the development and use of PFAS-containing AFFF. These highly effective foams would be used for years and lead to environmental and clean-up issues costing billions. The US Department of Defense (DOD) has been funding the development of fluorine-free foams (F3) since 2017. The DOD released specifications for F3 in January 2023, and was required to stop buying PFAS-containing foam by October 2023 and replace all AFFFs by October 2024 [222]. The U.S. military is currently transitioning to F3 products.

However, F3 and other green chemistry firefighting foams are not a direct replacement for PFAS-containing AFFF because they vary in performance for class B fire suppression across different fuel types [223]. Whatever the military decides and what alternatives the military deems best, based on its criteria of efficacy, safety, cost, and environmental health concerns, will affect local municipal and airport fire stations, forest services, chemical plants, oil refineries, oil tankers, and offshore platforms because these military-preferred foams will most likely become the new standard.

Switching from PFAS is not an easy task. There are several types of new synthetic firefighting foams to work with and learn, and each type may work differently from traditional AFFF or other modern non-PFAS synthetic foams. For example, the specific type of fire or size of the fire may impact the foam’s performance or preferred use. Some firefighting equipment may need to be modified or completely replaced to handle the new foams because of different application densities, proportioning rates, viscosities, and flows. How long the new synthetic foams last under variable storage conditions also needs to be considered. Lastly, firefighters will need training to learn how to properly use the new foams [222].

As PFAS-containing AFFF compounds are being phased out due to their toxicity and bioaccumulative potential, there is an urgent need to assess the bioaccumulation potential and toxicity of the PFAS-free AFFF replacement products. The Strategic Environmental Research and Development Program (SERDP) run by the DoD has been studying potential PFAS-free AFFF. Early data suggest that the PFAS-free AFFF products are predicted to have a much lower likelihood of environmental persistence and bioaccumulation [224,225]. For example, the toxicity of six replacement products was assessed: BIOEX ECOPOL A 3%, Fomtec Enviro USP, National Foam 20-391, National Foam Avio^F3^ Green KHC 3%, National Research Lab (NRL)-502W, and Solberg Re-Healing Foam RF3 3%. These PFAS-free alternatives were compared to Buckeye Platinum 3% PFAS-containing AFFFs and showed greater biodegradability. ToxPi 2.3, a free downloadable software package for prioritizing and visualizing data, was used to rank toxicity [226]. ToxPi indicates that BIOEX ECPOL was the least hazardous to human and animal health based on the hazard assessment criteria, and Solberg Re-Healing Foam RF3 3% was the most hazardous replacement product. Comparatively, the short-chain PFAS Buckeye Platinum 3% was the second-least hazardous [224].

A recent investigation of the biodegradability of those same six alternative PFAS-free AFFF formulations (BIOEX ECOPOL A 3%, Fomtec Enviro USP, National Foam 20-391, National Foam Avio^F3^ Green KHC 3%, National Research Lab (NRL)-502W, and Solberg Re-Healing Foam RF3 3%) revealed that most formulations had similar metabolite formation and were readily degraded within the 28-day trial [227]. Ecotoxicity studies indicate that F3 may exhibit similar or even greater toxicity to aquatic biota than AFFFs [223]. The acute toxicity of these six PFAS-free compounds and one PFAS-containing reference compound was evaluated with 14 freshwater, marine, and terrestrial species. These studies determined that exposure to some PFAS-free AFFF formulations can result in equal or greater acute toxicity in aquatic species compared to PFAS-containing AFFF [228]. Species-specific differences were noted, such as lower toxicity thresholds for estuarine invertebrates such as the mud snail (*Tritia obsoleta*) and the sheepshead minnow (*Cyprinodon variegatus*) than corresponding freshwater species [228].

Standard chronic toxicity tests with a suite of freshwater and marine species and the same set of alternative AFFF formulations revealed significant effects on growth (*Daphinia magna*, *Chironomus dilutus*, *Pimephales promelas*, and *Americamysis bahia*) and reproduction (*D. magna*, *Ceriodaphnia dubia*, *C. dilutus*) [229]. While in many cases the PFAS-free AFFF products exhibited greater toxicity than the reference legacy AFFF, it should be noted that observed effect thresholds were generally >1 ppm [229]. More comprehensive toxicological profiles of F3 formulations are needed to determine mechanisms of action and degradation pathways under variable exposure conditions and to further characterize chronic toxicity, reproductive toxicity, and endocrine disruption [50,223]. The good news is that while these compounds exert greater acute toxicity in some cases, they have much lower bioaccumulation potential and therefore are less likely to cause persistent effects.

The primary purpose for switching to alternatives is the environmental health issues and subsequent clean-up costs. There are 714 military installations that are being assessed for PFAS contamination. As of 30 June 2023, 466 installations have been assessed, of which 359 of these installations are proceeding with further testing and risk assessments per CERCLA processes. Sites identified as imminent risks to human health after the Relative Risk Site Evaluations (RRSEs) will be prioritized. The Department of Defense (DoD) will take immediate action (provide bottled water, water filters, municipal filtration systems, etc.) at any site with PFOA or PFOS at drinking water concentrations above the EPA’s former 70 pptr threshold set in 2016 [230]. The immediate costs of the PFAS clean-up obligations are estimated at USD 2.02 billion; however, this estimate is expected to increase as the DoD completes its initial assessments and determines what actions are required [231]. In 2021, it was estimated that the total costs of PFAS remediation at 50 contaminated military sites would be USD 3.7 billion; however, just 2 years later, and those estimates had ballooned to USD 31 billion. These are just the costs associated with military installations [232]. Therefore, it is unaffordable for most municipalities or states to clean up PFAS from wastewater. For example, Minnesota estimates its PFAS clean-up costs to approach USD 28 billion over the next 20 years [233]. Sadly, the new short-chain PFAS are more water-soluble and difficult to destroy, and therefore, it could be 70% more expensive to mitigate their contamination in water through conventional means such as activated carbon [233]. Thus, new and potentially more expensive methods may need to be developed. However, expectations are that some or all of them will have shorter half-lives, and this could reduce that concern.

Another way to reduce the use of PFAS-based AFFFis to reduce the potential for fires or limit the number of alcohol- and fuel-based fires. The move to electric vehicles (EVs) should continue to reduce fires despite the problems associated with certain EV models. Fires occur most often in hybrids with a frequency of 3475/100,000 vehicles. Fires occur in gas-powered vehicles at a frequency of 1530/100,000 vehicles, whereas fires occur in EVs at a frequency of 25/100,000 vehicles. This is an incredible 61X decrease from gas-powered vehicles [234]. It should be noted that EV fires can burn longer because of the Li-ion batteries and bring a new set of firefighting challenges. Included in these challenges is lithium bis ((trifluoromethyl)sulfonyl)imide (HQ-115), a more water-soluble PFAS that is considered safer than the lithium hexafluorophosphate that it replaced [235]. The USEPA has made a call for further research on the economic, environmental, and human health impacts of HQ-115 [236]. Current research data indicates that HQ-115 has hazardous effects on the kidney, testes, and liver; however, it is not as toxic as PFOA or PFOS [237,238,239]. Overall, the move to EVs and electric sources of power instead of liquid-ignition fuels could reduce the reliance on AFFF and alternative foams, providing a different means of reducing AFFF’ environmental contamination and adverse human health effects.

## 11. Conclusions

Significant knowledge gaps remain about the fate, exposure, and effects of PFAS, particularly on human and ecosystem health. There are even more gaps in the knowledge of the bioaccumulation and toxicity of PFAS-free formulations such as F3. Additional testing of PFAS alternative products (F3) will be beneficial for ensuring the human and environmental safety of replacement compounds. Very little research has addressed the chronic toxicity of the new AFFF on the market, also an area of research needed for legacy and emerging PFAS [50,223]. In addition, much PFAS research has evaluated exposure scenarios involving food or water resources; however, consumer products have been understudied and are an area that some states in the USA require further information [240,241]. In the USA, there are several state-specific guidelines that affect policies and provide policy challenges for drinking water, remediation, AFFF, consumer products, and food packaging [241,242,243].

Drawing from historical examples in the One Health literature, it will be crucial to move past conventional interdisciplinary barriers that separate human health, veterinary, ecological, environmental, and computational sciences to develop novel PFAS control and remediation strategies and reduce the risks that PFAS pose to human, animal, and ecosystem health [244]. To address these needs, future studies should prioritize the collection of physiochemical property data, determine effect-based toxicity values in a variety of matrices, and establish methods to effectively measure the bioaccumulation and biomagnification potential of PFAS-containing and PFAS-free AFFF formulations for the development of predictive models [57,245]. In addition, prospective and longitudinal human studies are indispensable, with a focus on the complex PFAS mixtures that reflect real-life exposure scenarios, to more definitively assess the health risks of legacy, emerging, and PFAS alternatives, which can help to prioritize investment in mitigation strategies and ecological health, sensitive populations, public health, and clinical interventions. These studies may help enable better regulation of specific classes of PFASbased on their properties, toxicity, and bioaccumulation.

## Data Availability

Not applicable.
